# Effect of low-level laser therapy (904 nm) and static stretching in patients with knee osteoarthritis: a protocol of randomised controlled trial

**DOI:** 10.1186/s12891-015-0709-9

**Published:** 2015-09-14

**Authors:** Sarah Rubia Ferreira de Meneses, David John Hunter, Eunice Young Docko, Amelia Pasqual Marques

**Affiliations:** Department of Physiotherapy, Occupational Therapy and Speech Therapy, School of Medicine, University of Sao Paulo, Sao Paulo, Brazil; Royal North Shore Hospital, Rheumatology Department, and Institute of Bone and Joint Research, Kolling Institute, University of Sydney, Sydney, NSW Australia

## Abstract

**Background:**

Osteoarthritis (OA) is a highly prevalent and disabling disease. It is estimated that by 2030 the prevalence of symptomatic OA could reach 30 % of the population above 60 years. This randomised controlled trial will investigate the effect of low-level laser therapy (LLLT) and static stretching exercises, as monotherapy and in combination, on pain, quality of life, function, mobility, knee range of motion (KROM) and hamstring shortening in participants with knee OA.

**Methods:**

This study will involve 145 people aged 50–75 years with symptomatic-radiographic knee OA. It will consist of two types of treatments: Low-level laser therapy (LLLT) and stretching exercises. The patients will be randomly allocated to five groups LLLT_ACTIVE_+Stretch, LLLT_PLACEBO_+Stretch, Stretch, LLLT and Control (*n* = 29 each). Treatment frequency will be three sessions/week for all active groups. LLLT will involve the use of a Gallium-Arsenide laser (904 nm, 40 milliwatts, 3 J/point, 27 J/knee) over 24 sessions for the monotherapy group and 9 sessions for the LLLT+Stretch groups. Stretching will consist of seven exercises completed over 24 sessions. The control group will receive a booklet. Participants will be treated for 2 months (Stretch, LLLT and Control groups) or 3 months (LLLT + Stretch groups). Participants and the outcome assessor will be blind to treatment allocation throughout the study.

The primary outcome is pain measured by Visual Analogue Scale. Secondary outcomes include quality of life assessed by Western Ontario and McMaster Universities Arthritis Index, function by Lequesne Algofunctional Index, mobility by Timed Up and Go Test, KROM by goniometry of knee flexion and hamstring shortening by popliteal angle. The statistical method will follow the principles of per-protocol analysis.

**Discussion:**

Although exercise therapy is considered an effective treatment in patients with knee osteoarthritis, the knowledge of which exercise modalities would be the most appropriate for this population is lacking. LLLT has been used as resource to increase the effects of physical therapy. However, the specific dose and treatment frequency need to be better defined. The findings from this randomised controlled trial will provide evidence of the efficacy or otherwise, of LLLT and stretching exercises in the management of knee OA symptoms.

**Trial registration:**

NCT01738737 at ClinicalTrials.gov.

## Background

Osteoarthritis (OA) is the most common form of arthritis [1] and is a leading cause of global disability [2, 3]. The disease occurs when the dynamic equilibrium between deterioration and restoration becomes unbalanced, often in situations where the mechanical stress applied is greater than the one that can be supported by the joint tissues. OA is typically a progressive disease of the whole synovial joint characterized by progressive loss of cartilage, remodelling of subchondral bone, osteophytes and synovitis. The peri articular tissues are also affected from the disease, resulting in muscle atrophy and ligament dysfunction. As a consequence of these changes, joint pain and functional disability may ensue [4].

It is estimated that 10 % of the population above 60 years have medical problems that can be attributed to OA [5]. It is estimated that by 2030, the prevalence of symptomatic OA will reach 30 %; attributed to increasing life expectancy and the rising number of persons with obesity [6]. Knee OA (KOA) accounts for a significant portion of affected people. Pereira et al. [7] estimated the prevalence of radiographic KOA for all age groups and found a value of 27.3 % for women and 21.0 % for men.

Clinical manifestations include joint pain, stiffness, decreased range of motion (ROM), muscle weakness, proprioceptive changes [8], difficulties in activities of daily living (ADL) such as walking, climbing / descending stairs and housekeeping [9]. Deformities and instabilities can also be observed. Joint pain is the dominant symptom, accentuated when the joint is moved and relieved with rest. Persistent pain even during rest or at nocturnal rest may be a sign of advanced OA [10]. Pain and stiffness are the two primary reasons for ADL and mobility disability, adversely affecting the quality of life of patients with KOA [11] and their seeking medical attention.

Management is typically focused on symptom control and in the first instance conservative non-pharmacologic management (e.g. weight loss, exercise) is advocated by most therapeutic guidelines. Physiotherapy treatment aims to relieve pain, improve function, quality of life, mobility, joint function, knee stabilization, reduce the load on the joint, promote adaptation of certain activities, prevent deformities and slow the progression of the disease [12]. Jamtvedt et al. [13] conducted an analysis of systematic reviews related to physical therapy for KOA and found that only exercise and weight reduction had high quality evidence on improvement in pain and function. Acupuncture, transcutaneous electrical nerve stimulation (TENS) and Low Level Laser Therapy (LLLT) had moderate quality of evidence for the same variables. Other interventions achieved a quality rating of low or non-existent evidence.

Recent meta-analysis on the effect of LLLT on KOA concluded that if administered within optimum dose levels and under a treatment regimen of 2–4 weeks, affords clinically relevant pain relief over placebo [14]. However, a systematic review showed conflicting results [15]. One potential reason for this conflict pointed out by the authors is the great variability in relation to the wavelength, dosage, localization of application points, frequency and duration of treatment and the absence of calibration of the laser.

LLLT has been used successfully to control pain of various musculoskeletal disorders [16–18]. In view of the inflammatory nature of OA, it is believed that laser treatment can play a beneficial effect by modulating the inflammatory process. However, the literature indicates inconsistent results in reducing pain in persons with KOA; one explanation for this inconsistency is the lack of studies that specify what dosage and frequency should be used. In our opinion, LLLT is still underutilized and as our study proposes to test the dosage indicated by the World Association for Laser Therapy (WALT) [19], it is expected that the results of this research may provide the foundation for evidence based clinical practice.

Persons with KOA tend to avoid physical activity in an attempt to prevent pain. It was shown that this behaviour, over time, reduces the strength of knee extensors further impairing ability to perform activities of daily living [20]. When subjected to immobilization or inactivity, the peri articular connective tissue becomes fibrotic, resulting in capsular adherence, adaptive shortening of muscles and consequent limitation of ROM [21] . Studies have reported that individuals with KOA have increased hamstring muscle activity, preventing the joint reaching its maximum extension during the gait cycle, a fact that further compromises knee ROM [22, 23]. Pain and reduced ROM in affected joints are important risk factors for the occurrence of functional disability [24]. It is presumed that stretching performed slowly and sustainably must be performed to change this picture.

Therapeutic exercises have proven to be beneficial for individuals with KOA, however it is unclear which type, frequency and intensity are more suitable. We propose to test muscle stretching in this study in order to ascertain if this type of exercise is appropriate for this population. Despite their relevance, studies testing stretching exercises are scarce.

The combination of these two treatments (LLLT and stretching) aims to relieve pain with the initial use of the laser to enhance the effect and implementation of stretching exercises. Thus, the hypothesis of this study was that LLLT combined with stretching exercises would demonstrate greater benefits for patients with KOA than either treatment alone.

The primary objective of this research will be to investigate the effect of static stretching and LLLT (904 nm), in combination or as monotherapy, in the management of pain in persons with KOA. The secondary objective was to evaluate the influence of each intervention on quality of life, function, mobility, knee flexion range of motion and hamstring length of these individuals compared to a control group.

## Methods

### Trial design

This study will be a five-arm randomised controlled trial with blind evaluator.

### Approval and registration

The procedures and consent form were approved by the Research Ethics Committee of Faculty of Medicine of the University of Sao Paulo (protocol no. 455/11). The study is being funded by *Fundação de Amparo à Pesquisa do Estado de São Paulo* (FAPESP) (2012/01827–3), *Coordenação de Aperfeiçoamento de Pessoal de Nível Superior* (CAPES) (Institutional) and *Conselho Nacional de Desenvolvimento Científico e Tecnológico* (CNPq) (248967/2013-4). This study was registered at ClinicalTrials.gov - NCT01738737.

### Participants

One hundred and forty-five men and women aged 50–75 years with symptomatic radiographic knee OA will be recruited from the *Serviço Especializado em Reabilitação* (SER) of Taboao da Serra, Sao Paulo, Brazil. This clinic provides multidisciplinary care, with specialties in orthopaedics, rheumatology, neurology, psychology, physiotherapy, occupational therapy, speech therapy, nursing and social care. The use of this site was authorized by the Secretary of Health of Taboao da Serra and by the General Director of the establishment.

Participants will be eligible if they have (i) radiographic evidence of knee osteoarthritis between 2 and 4 in Kellgren and Lawrence classification [25]; (ii) pain intensity ≥ 3 on a 10 cm Visual Analogue Scale (VAS); and (iii) knee symptoms for at least 3 months.

Participants will be ineligible if they (i) have symptomatic hip osteoarthritis; (ii) have any disease where laser treatment is contraindicated (cancer and uncontrolled diabetes mellitus); (iii) use continuous anti-inflammatory drugs; and (iv) have other concurrent injury/conditions that will affect their ability to participate in the rehabilitation program and/or assessment procedures.

All participants will be informed about the study’s objectives, timeline, and eligibility criteria, then asked to sign an informed consent form if they agree to take part in the study. Participants excluded at this stage will be referred to the physiotherapy sector and followed with the standard of care provided by the service (10 sessions).

### Study procedure

All participants will be screened by an orthopaedist or rheumatologist to diagnose and identify the severity of KOA. The diagnosis will be based on the radiograph analysis and physical assessment and the severity will follow the Kellgren & Lawrence classification. After confirmation of KOA patients will be referred to the local physiotherapy sector. The eligibility criteria will be applied during the prior assessment standardized by the clinic, and when the patient is considered eligible and is interested in participating in the study, their contact (full name and phone number) will be passed to the study assessor in order to schedule the baseline assessment.

Participants will be instructed to bring knee radiographs, prescription medications taken and the referral for physical therapy. Participants will be asked to use comfortable clothes and not to use analgesic or anti-inflammatory drugs 48 h prior the evaluation date, but to continue to perform their ADLs in the usual way. On the baseline visit, the criteria for inclusion and exclusion will be reapplied.

Five intervention groups will be part of the study:

- LLLT_ACTIVE_ + Stretch: 3 weeks of low intensity laser therapy (LLLT) followed by 8 weeks of stretching exercises.
- LLLT_PLACEBO_ + Stretch: 3 weeks of placebo LLLT followed by 8 weeks of stretching exercises.
- Stretch: 8 weeks of stretching exercises.
- LLLT: 8 weeks of active LLLT.
- Control: minimal intervention through educational booklet.

This study will be conducted by a trained physiotherapist.

### Assessment procedures

Participants will be assessed prior to starting treatment and after each intervention. The initial part of the evaluation will consist of a socio-demographic questionnaire, which includes personal information, medical history, history of past and present illness, diseases and medications taken. Participants who continuously use NSAIDs and / or medications to control OA (glucosamine sulphate and chondroitin) will be instructed to discontinue the drug until the end of the trial to avoid possible research bias. The suspension of these drugs will be with the prior knowledge of their physicians, since the study was approved by the General Board of the SER. For pain control during the intervention period, only analgesics (e.g. paracetamol, dipyrone) and thermotherapy will be allowed and their use recorded, but these methods will be interrupted 48 h prior periods of reassessment in order to avoid possible confounders.

The following instruments and tests will be used to assess the participants: (i) Western Ontario and McMaster Universities Arthritis Index (WOMAC); (ii) Lequesne index for knee osteoarthritis; (iii) VAS; (iv) goniometry of knee range of motion (KROM); (v) popliteal angle; and (vi) Timed Up and Go (TUG). This data will be collected for the knee with greater severity of symptoms. All scales and questionnaires have been translated and cross-culturally adapted to the Brazilian population. A detailed description of each of the instruments is given below.

- Western Ontario and McMaster Universities Arthritis Index: The questionnaire contains 24 questions of which 5 evaluate pain, 2 joint stiffness and 17 function [26, 27]. Each question is graded qualitatively, with the response options: none, low, moderate, severe and very severe. The equivalent scores are 0, 1, 2, 3 and 4, respectively. Higher scores indicate greater impact on quality of life. Although self-administered the questionnaire will be verbalized and filled by the assessor in an attempt to standardize the level of understanding of the subject and to minimize filing errors.
- Lequesne Index: is composed of 11 questions about pain, discomfort and function [28, 29]. One of the items is specific for hip OA, so this question will be deleted. Each answer has an equivalent score. The total score ranges from 0 to 24 and is divided into five categories of functional impairment: no = 0, bit = 1–4, moderate = 5–7, severe = 8–10, very severe = 11–13 and extremely severe ≥ 14. In conclusion, the higher the score, the greater the impairment of function.
- Visual Analogue Scale: The VAS has been shown to be a reliable and valid measure of pain, which is used frequently in clinical and research settings [30]. It consists of a 10-cm line anchored at each end. The left-hand anchor reads ‘no pain’ and the right-hand anchor reads ‘worst possible pain’; the participant marks a line to represent their pain level. Pain during movement and rest will be measured. A ruler will measure the distance in centimetres from the beginning of the line to the marked point, with higher values representing more severe pain.
- Goniometry of knee range of motion: will be measured with a universal goniometer (CARCI). Active extension will be measured with the participant in a supine position with extended legs. For goniometry of active flexion, the participant will be in the prone position with the contralateral lower limb in extension. The KROM will be calculated as follows: angle of active flexion less the angle of active extension. The measures will follow the methodology of Marques [31].
- Popliteal angle: will be measured with a universal goniometer. The patient will be positioned in supine position with a ratio of 90° of hip and knee flexion and with the contralateral limb extended. From that position, without losing the hip flexion, the knee will be passively stretched to the limit (joint or tissue). The amount remaining to full extension represents the popliteal angel.
- Timed Up and Go: this test has been shown to be reliable, reproducible, and responsive to change [32]. Participants will be asked to rise from a standard armchair, walk at a safe and comfortable pace to a mark 3 m away, then return to a sitting position in the chair, using gait aids and chair armrests to assist with sit to stand as needed. The outcome measured will be the time to complete the task. The longer the time spent, the worse their mobility.

### Interventions

All monotherapy groups had durations of 8 weeks, as this duration is usually used in knee osteoarthritis clinical trials as an effective period of treatment [33]. Additionally, this study includes two supplementary groups with a preparatory period of 3 weeks of laser active/placebo before the standard 8 weeks of exercise treatment. The World Association of Laser Therapy suggests a 2–3-week duration for laser therapy [19]. A potential group involving simultaneous laser/placebo and stretching exercises, within a single 8-week period, was not considered as the number of treatments per week and the time spent would not be feasible with clinical practice. If the chosen preparation period is found to improve the standard 8-week period of exercise then further studies could determine the optimal combination of treatment durations. 

#### Low-level laser therapy (LLLT)

The LLLT will be performed using pulsed laser, class 3B and wavelength of 904 nm. The equipment will be the LASERPULSE manufactured by IBRAMED (Brazil) and with a Gallium Arsenide (GaAs) diode laser, which has the following technical characteristics: peak power 70 W; pulse duration 60 ns; pulse repetition rate 9500 Hz and beam area 0,1 cm^2^. Detailed information present in Table 1.

**Table 1:** Device information, irradiation and treatment parameters

Device information		
Manufacturer	IBRAMED	
Model identifier	LaserPulse	
Number of emitters	1	
Emitter type	GaAS	
Beam delivery system	Hand-held probe	
Irradiation parameters		
Parameter [unit]	Value	
Center wavelength [nm]	904	
Spectral bandwidth [nm]	724 – 1009	
Operating mode	pulsed	
Frequency [Hz]	9,500	
Pulse duration [ns]	60 ns ± 20 %	
Peak radiant power [W]	70	
Average radiant power [mW]	40	
Beam profile	Circular	
Treatment parameters		
Parameter [unit]	Value	Additional notes
Beam spot size at target [cm2]	0.13090	
Irradiance at target [mW/cm2]	305.6	
Exposure duration [sec]	11 min and 25 s	Per knee
Radiant exposure [J/cm2]	22.9	
Radiant energy [J]	3	Per knee
Number of points irradiated	9 points per knee	
Area irradiated [cm2]	0.13090	Per point
Application technique	Contact with pressure	
Number and frequency of treatment sessions	9 treatments total - 3×/week	
Total radiant energy [J]	27	Per knee

The recommendations of the World Association for Laser Therapy (WALT) for the treatment dose in KOA will be respected. Thus, the dose of 3 J(J)/point will be used. Real average power and exposure time were calculated based on the study of Fukuda & Malfatti [34]. Therefore, the power for this device is 40 milliwatts and the time required for the dose of 3 J will be 75 s(s) per point. The probe and the laser device will be checked regularly (every 6 months) to ensure proper function.

**Fig. 1:**
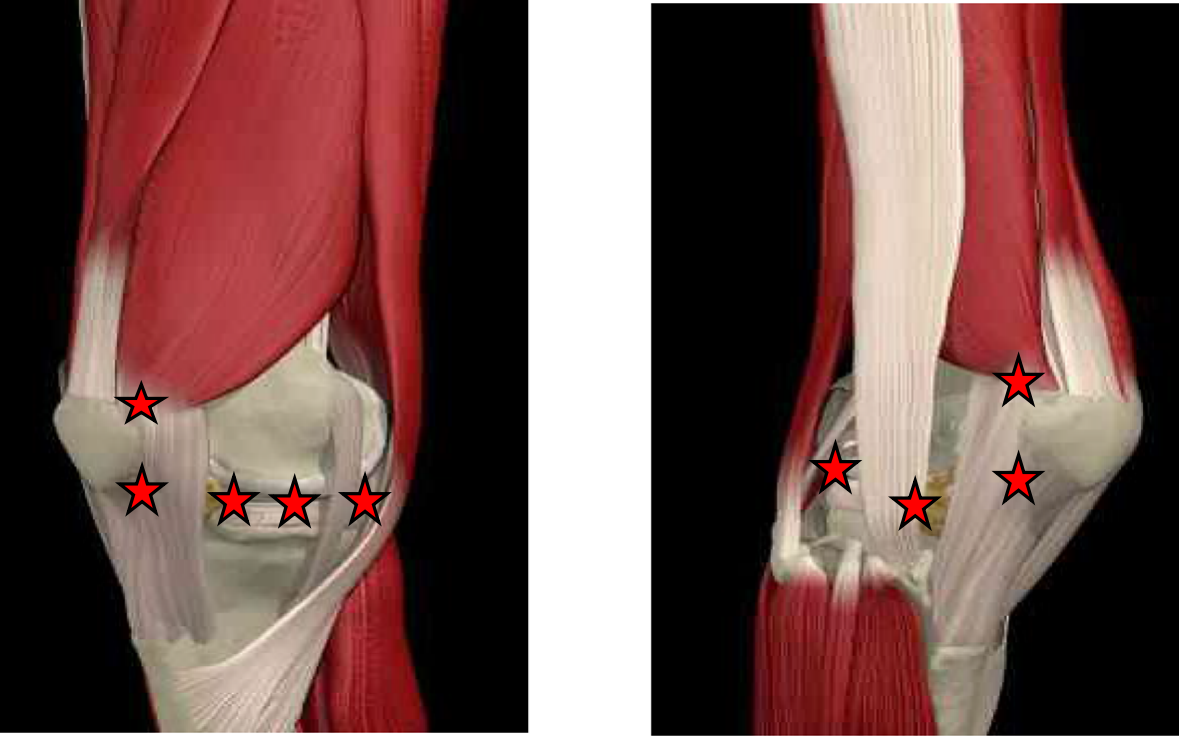
Application points of LLLT

Nine knee points will be irradiated with LLLT: three on the medial joint line; two on the lateral joint line and four points over the borders of kneecap, as shown in Fig. 1 - Application points of LLLT. Each point will receive energy of 3 J/point for 75 s, with a total dose of 27 J/knee in each session. Thus, the time of application will be approximately 12 min/knee. All participants and the physiotherapist will wear safety goggles to shield their eyes from active laser radiation. For participants in the placebo group, the treatment procedure will be identical but without switching on the machine.

Participants will assume a sitting position and the laser probe will be placed on the knee points sequentially and perpendicularly and in full contact with the skin. All participants will attend the LLLT sessions three times per week over a period of 3 weeks in the combined treatment groups and for 8 weeks when used as monotherapy.

#### Muscle stretching exercises

Before running stretching exercises, patients will hold a 10-min period for warming up on a stationary bike with light load (3/9) and comfortable speed. However, those unable to use this machine will warm-up on a treadmill at a speed of 2.0 km/h and no slope.

The treatment will be conducted in groups of five to seven people at a frequency of 3×/week and lasting approximately 45 min. The intervention will last 8 weeks, completing 24 sessions and will consist of seven segmental stretching exercises, repeated four times and sustained for 30 s each. The intensity will follow the recommendation of the American College of Sports Medicine [35] positioning with middle discomfort. Unilateral exercises are performed alternately, allowing the contralateral limb to rest during execution. For bilateral exercises, a rest period of 30 s between the repetitions will be established. Participants will be correctly positioned and the responsible physiotherapist will guide body awareness, breathing and alignment throughout the therapy. After completing each exercise, individuals will adopt a posture of rest.

**Fig. 2 Muscle stretching exercises:**
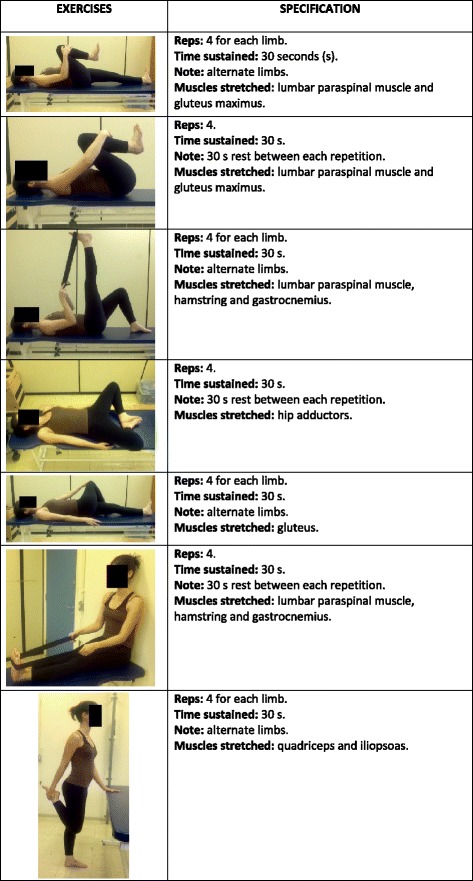
ᅟ

The major muscles of the posterior and antero-internal hip muscle chains will be stretched: paraspinal muscle, gluteus, iliopsoas, hamstrings, quadriceps, hip adductors and gastrocnemius (Fig. 2 – Stretching exercises).

#### Control

The control group will receive an educational booklet at the baseline visit consisting of explanations about the disease, postures during daily activities and information for the management of pain. The physiotherapist will explain in detail each item and make weekly telephone follow-up questioning about doubts regarding the educational content and the participants’ state of health. Reassessment will occur after 8 weeks.

### Randomisation and allocation concealment

The randomisation schedule will be prepared by a researcher not involved in participant recruitment, assessment or treatment using a computer-generated random numbers table. There will be a 1:1 allocation ratio of participants to each one of the five groups. Randomisation will be conducted by random permuted blocks of same size. Each group will have 29 numbers. The list containing this information will remain with the physiotherapist responsible for the treatment.

A number will be assigned to participants according to the order of their evaluation. This digit will be later located in the list and, thus, the corresponding group will be identified. The assessor will perform the assessment unaware of the group to which each participant will belong. After the initial evaluation, the participant will be informed by the physiotherapist responsible of the type, time and frequency of treatment.

### Statistical analysis

All data will be submitted to a descriptive inferential analysis. For adherence to normality, the Kolmogorov-Smirnov test will be conducted. The intergroup comparison will be performed by simple variance analysis (one-way ANOVA) for data with normal distribution and the ANOVA Kruskal-Wallis for non-normal distribution. For statistically significant differences, post hoc tests like Holm-Sidak (parametric) and Tukey test (nonparametric) will be applied. Categorical variables will be compared by chi-square test.

In view of the need to determine clinically important differences, we will calculate the Relative Gain that corresponds to the gain that the patient obtained after the end of treatment relative to how much they could improve. The Average Gain will be calculated for intergroup analysis and will be obtained by the difference between pre and post intervention for each variable. The data will be processed by the software SigmaStat 3.5. The significance level for all analyses will be α = 0.05.

### Sample size calculation

The primary outcome measure VAS (range: 0–10 cm) was used to estimate the sample size. Using a minimal clinically important change of two-point difference between the treatment groups and control group, with a significance level of 0.05 (2-tailed) and a power of 80 %, we estimated that 25 participants were needed in each group. To allow for a 15 % drop-out rate, we aimed to include 29 participants in each group.

### Timeline

Ethics approval was obtained in December 2011 from the Research Ethics Committee of Faculty of Medicine of the University of Sao Paulo (protocol n° 455/11). Recruitment and training of the assessor was carried out in May 2012. Recruitment of participants commenced in June 2012. All participants completed the study in December of 2014. We aim to finish the statistical analysis by the end of August of 2015 and after that start the elaboration of scientific papers.

## Discussion

This paper has presented the protocol for an ongoing randomised controlled trial (RCT) to investigate the effect of low-level laser therapy (LLLT) and static stretching exercises, as monotherapy and in combination, on pain, quality of life, function, mobility, knee range of motion and hamstring length in participants with KOA.

The findings of the study will provide reliable information that will guide the future use of LLLT in patients with KOA since the American Academy of Orthopaedic Surgeons (AAOS) guideline was unable to recommend for or against the use of physical agents [36]. Regardless of the whether the findings of the present study are positive, neutral or negative, the results will be important for guiding evidence based practice. For example, if adding LLLT to muscle stretching program provides greater pain relief and functional improvement than stretching exercises alone, this method could be advised as an effective treatment option for these patients. Conversely, if LLLT does not provide additional incremental or singular benefit then this widely practiced intervention, would have evidence to discourage that practice.

Guidelines and meta-analyses request more RCTs evaluating type, intensity, and frequency of exercise, which may contribute to the knowledge of dose–response relationship as well as to the efficacy of stretching as an exercise modality in persons with knee OA. The European League Against Rheumatism (EULAR) recommendations indicate stretching exercises as an adjunctive treatment [37]. However, to our knowledge, no studies have verified the effectiveness of stretching as monotherapy. This study will contribute with evidences of the use of this modality in clinical practice and will provide additional insights for the conservative treatment options.

It is important to note that the ethics committee did not approve the inclusion of a group with placebo laser as monotherapy, resulting in a study limitation.

## Abbreviations

OA: Osteoarthritis
KOA: Knee osteoarthritis
LLLT: Low-level laser therapy
ADL: Activities of daily living
KROM: Knee range of motion
TENS: Transcutaneous electrical nerve stimulation
WALT: World Association for Laser Therapy
FAPESP: *Fundação de Amparo à Pesquisa do Estado de São Paulo*
CAPES: *Coordenação de Aperfeiçoamento de Pessoal de Nível Superior*
CNPq: *Conselho Nacional de Desenvolvimento Científico e Tecnológico*
SER: *Serviço Especializado em Reabilitação*
VAS: Visual analogue scale
WOMAC: Western Ontario and McMaster Universities Arthritis Index
TUG: Timed up and go
GaAs: Gallium arsenide
EULAR: European league against rheumatism
AAOS: American academy of orthopaedic surgeons
RCT: Randomised controlled trial

## References

[1] Lawrence RC, Felson DT, Helmick CG, Arnold LM, Choi H, Deyo RA (2008). Estimates of the prevalence of arthritis and other rheumatic conditions in the United States. Part II. Arthritis Rheum.

[2] Vos T, Flaxman AD, Naghavi M, Lozano R, Michaud C, Ezzati M  (2012). Years lived with disability (YLDs) for 1160 sequelae of 289 diseases and injuries 1990–2010: a systematic analysis for the Global Burden of Disease Study 2010. Lancet.

[3] Murray CJ, Vos T, Lozano R, Naghavi M, Flaxman AD, Michaud C (2012). Disability-adjusted life years (DALYs) for 291 diseases and injuries in 21 regions, 1990–2010: a systematic analysis for the Global Burden of Disease Study 2010. Lancet.

[4] Hunter DJ (2011). Osteoarthritis. Best Pract Res Clin Rheumatol.

[5] Woolf A, Pfleger B (2003). Burden of major musculoskeletal conditions. Bull World Health Organ.

[6] Croft P (2005). The epidemiology of osteoarthritis: Manchester and beyond. Rheumatology (Oxford).

[7] Pereira D, Peleteiro B, Araújo J, Branco J, Santos RA, Ramos E (2011). The effect of osteoarthritis definition on prevalence and incidence estimates: a systematic review. Osteoarthr Cartil.

[8] Kaufman KR, Hughes C, Morrey BF, Morrey M, An KN (2001). Gait characteristics of patients with knee osteoarthritis. J Biomech.

[9] Bennell KL, Hunt MA, Wrigley TV, Hunter DJ, Hinman RS (2007). The effects of hip muscle strengthening on knee load, pain, and function in people with knee osteoarthritis: a protocol for a randomised, single-blind controlled trial. BMC Musculoskelet Disord.

[10] Michael JWP, Schlüter-Brust U, Eysel P (2010). The epidemiology, etiology, diagnosis, and treatment of osteoarthritis of the knee. Dtsch Arztebl Int.

[11] Hunt MA, Keefe FJ, Bryant C, Metcalf BR, Ahamed Y, Nicholas MK (2013). A physiotherapist-delivered, combined exercise and pain coping skills training intervention for individuals with knee osteoarthritis: a pilot study. Knee.

[12] Huleatt JB, Campbell KJ, La Prade RF (2014). Nonoperative treatment approach to knee osteoarthritis in the master athlete. Sports Health.

[13] Jamtvedt G, Dahm KT, Christie A, Moe RH, Haavardsholm E, Holm I (2008). Physical therapy for patients with osteoarthritis of the knee: an overview of systematic reviews. Phys Ther.

[14] Bjordal JM, Johnson MI, Lopes-Martins RA (2007). Short-term efficacy of physical interventions in osteoarthritic knee pain: a systematic review and meta-analysis of randomized placebo controlled trials. BMC Musculoskeletal Disord.

[15] Brosseau L, Welch V, Wells G, DeBie R, Gam A, Harman K (2004). Low level laser therapy (Classes I, II, and III) for treating osteoarthritis. Cochrane Database Syst Rev.

[16] Hegedus B, Viharos L, Gervain M, Galfi M (2009). The effect of low-level laser in knee osteoarthritis: a double blind, randomized, placebo-controlled trial. Photomed Laser Surg.

[17] Ozdemir F, Birtane M, Kokino S (2001). The clinical efficacy of low power laser therapy on pain and function in cervical osteoarthritis. Clin Rheumatol.

[18] Simunovic Z, Trobonjaca T, Trobonjaca Z (1998). Treatment of medial and lateral epicondylitis—tennis and golfer’s elbow—with low laser therapy: a multicenter double-blind, placebo-controlled study on 324 patients. J Clin Laser Med Surg.

[19] World Association of Laser Therapy. Recommended treatment doses for low level laser therapy. [http://waltza.co.za/wp-content/uploads/2012/08/Dose_table_904nm_for_Low_Level_Laser_Therapy_WALT-2010.pdf]. Accessed 9 August 2015.

[20] Pisters MF, Veenhof C, van Dijk G, Dekker J (2014). Avoidance of activity and limitations in activities in patients with osteoarthritis of the hip or knee: a five year follow up study on the mediating role of reduced muscle strength. Osteoarthritis Cartilage.

[21] Weng MC, Lee CL, Chen CH, Hsu JJ, Lee WD, Huang MH (2009). Effects of different stretching techniques on the outcomes of isokinetic exercise in patients with knee osteoarthritis. Kaohsiung J Med Sci.

[22] Messier SP, Loeser RF, Hoover JL, Semble EL, Wise CM (1992). Osteoarthritis of the knee: effects on gait, strength and flexibility. Arch Phys Med Rehabil.

[23] Hortobagyi T, Westerkamp L, Beam S, Moody J, Garry J, Holbert D (2005). Altered hamstring-quadriceps muscle balance in patients with knee osteoarthritis. Clin Biomech.

[24] Dekker J, van Dijk GM, Veenhof C (2009). Risk factors for functional decline in osteoarthritis of the hip or knee. Curr Opin Rheumatol.

[25] Kellgren JH, Lawrence JS (1957). Radiological assessment of osteoarthrosis. Ann Rheum Dis.

[26] Bellamy N, Buchanan WW, Goldsmith CH, Campbell J, Stitt LW (1988). Validation study of WOMAC: a health status instrument for measuring clinically important patient relevant outcomes to antirheumatic drug therapy in patients with osteoarthritis of the hip or knee. J Rheumatol.

[27] Fernandes MI, Ferraz MB, Ciconelli RM (2003). Tradução e validação do Questionário de Qualidade de Vida Específico para Osteoartrose (WOMAC) para a língua portuguesa. Rev Paulista Reumatol.

[28] Lequesne MG (1997). The algofunctional indices for hip and knee osteoarthritis. J Rheumatology.

[29] Marx FC, Oliveira LMD, Bellini CG, Ribeiro MCC (2006). Tradução e validação cultural do questionário algofuncional de Lequesne para OAJs e quadris para a língua portuguesa. Rev Bras Reumatol.

[30] Revill SI, Robinson JO, Rosen M, Hogg IJ (1976). The reliability of a linear analogue for evaluating pain. Anesthesia.

[31] Marques AP (2014). Manual de Goniometria.

[32] Piva SR, Fitzgerald GK, Irrgang IJ, Bouzubar F, Starz TW (2004). Get up and go test in patients with knee osteoarthritis. Arch Phys Med Reabil.

[33] Bennell KL, Dobson F, Hinman RS (2014). Exercise in osteoarthritis: moving from prescription to adherence. Best Pract Res Clin Rheumatol.

[34] Fukuda TY, Malfatti CA (2008). Análise da dose do laser de baixa potência em equipamentos nacionais. Rev Bras Fisioter.

[35] Pollock ML, Gaesser GA, Butcher JD, Despres J-P, Dishman RK, Franklin BA, Garber CE (1998). American College of Sports Medicine Position Stand: the recommended quantity and quality of exercise for developing and maintaining cardiorespiratory and muscular fitness, and flexibility in healthy adults. Med Sci Sports Exerc.

[36] Jevsevar DS (2013). American Academy of Orthopaedic Surgeons. Treatment of osteoarthritis of the knee: evidence-based guideline, 2nd edition. J Am Acad Orthop Surg.

[37] Fernandes L, Hagen KB, Bijlsma JWJ, Andreassen O, Christensen P, Conaghan PG (2013). EULAR recommendations for the non-pharmacological core management of hip and knee osteoarthritis. Ann Rheum Dis.

